# Platelet-rich Plasma-induced Inhibition of Chondrocyte Apoptosis Directly Affects Cartilage Thickness in Osteoarthritis

**DOI:** 10.7759/cureus.6050

**Published:** 2019-11-01

**Authors:** Rafia Asjid, Tayyaba Faisal, Khadija Qamar, Saleem Ahmed Khan, Aamna Khalil, Muhammad Sarwar Zia

**Affiliations:** 1 Anatomy, Army Medical College, Rawalpindi, PAK; 2 Haematology, Army Medical College, Rawalpindi, PAK; 3 Anatomy, Rawal Institute of Health Sciences, Islamabad, PAK; 4 Anatomy, Rawalpindi Medical University, Rawalpindi, PAK

**Keywords:** chondrocytes, apoptosis, osteoarthritis, cartilage

## Abstract

Abstract

The search for minimally invasive treatment of osteoarthritis has led to the development of biological options such as platelet-rich plasma (PRP), mesenchymal stem cells (MSCs), and bone marrow aspirate concentrates. This research was conducted to study the outcomes of PRP administration in the chemical-induced model of osteoarthritis in rat knee.

Methods and results

Two milligrams of monoiodoacetate (MIA) was used for the induction of arthritis in the right knee of 16 rats. Autologous PRP was prepared by double centrifugation, which was then administered in the arthritic knee of eight rats. This group was labeled as the treated group (A) while the rest were counted as the non-treated group (B). Chondrocyte count and uncalcified cartilage thickness were morphometrically assessed on hematoxylin and eosin (H&E) stained slides, and it was noted that treated group A had a higher chondrocyte count and more cartilage height as compared to non-treated group B. Intergroup comparison was done between the treated group (A) and non-treated group (B) using the independent t-test. P-values were found to be statistically significant for these parameters.

Conclusion

This study thus concluded that PRP had induced an inhibitory effect on the apoptosis of chondrocytes, which, in turn, prevented the loss of cartilage height by inhibiting matrix loss.

## Introduction

Osteoarthritis (OA) is a major source of disability, pain, and economic burden worldwide. Genetic, biochemical, and mechanical factors are responsible for the complex multifactorial epidemiology of the disease. Abnormal joint biomechanics, age, gender, joint injury, and high body mass index (BMI), along with a strong genetic basis, are associated with OA development. Presently, OA is the eighth-most common disease in males world over and the fourth most common disease in females [1].

Previously, OA was believed to be caused by the mechanical degradation of cartilage but recent advances in understanding the pathophysiology have led to the conclusion that it is a complex process in which matrix proteases play a fundamental role [2]. Type II collagen fibers constitute the main structural protein of cartilage, providing a latticework that is strengthened by other collagen types and proteins. Chondrocytes, which maintain the equilibrium between the catabolic and anabolic activities within the matrix, under inflammatory circumstances, are incapable of tolerating the insult and lead to the production of matrix metalloproteinases (MMPs), nitric oxide (NO), and prostaglandins (PGs), leading to matrix degradation [3]. The catabolic effects of interleukins secreted by chondrocytes, mononuclear cells, osteoblasts, and synovial cells interfere with the activity of growth factors and reduce the synthesis of aggrecan, which is the key constituent of the matrix providing resilience to cartilage [4]. Interleukin-1β (IL-1β), the proinflammatory cytokine, is a major protagonist in inducing arthritic changes, as evident by its increased levels in the synovial fluid of affected joints [5-6].

Recently, PRP has gained popularity due to its beneficial role in dental implant procedures, sports medicine, and orthopedics. When activated by exogenous agents, it releases mediators and growth factors, which are capable of limiting the inflammatory response and promote the healing of tissues. In the available in vitro and animal studies, PRP was found to promote the proliferation of chondrocytes, which have an anabolic effect on proteoglycan and collagen type II synthesis [7]. Studies conducted on mouse models have shown that PRP injections reduce pain and synovial membrane thickness [8]. Clinical trials have been carried out on hip and knee osteoarthritis, which showed an improvement in the WOMAC (Western Ontario and McMaster Universities Arthritis Index) and Harris scores for pain and function, respectively, but the exact role of PRP needs to be established [9]. This study was, therefore, carried out to observe the effects of intra-articular PRP injections on histological changes in the arthritic rat knee model.

## Materials and methods

Platelet-rich plasma releasate

**Figure 1:**
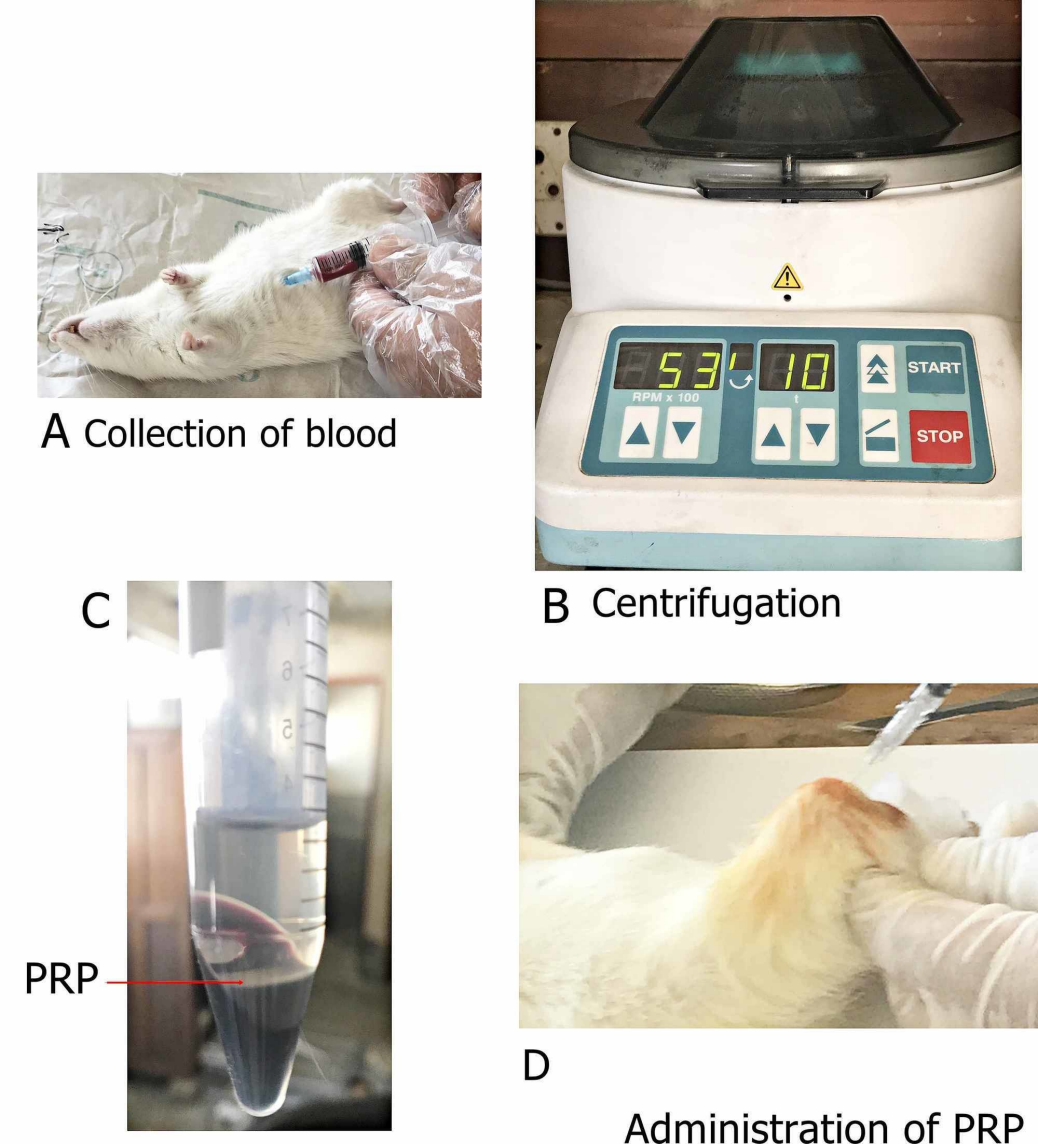
Figure showing various steps during the preparation of PRP A shows the collection of blood through the intracardiac route; B showing the process of centrifugation; C showing prepared platelet-rich plasma; and D showing the intra-articular administration of PRP PRP: platelet-rich plasma

Autologous PRP was prepared by taking 3 ml of blood in syringes containing 0.1 ml of sodium citrate. The intra-cardiac route of blood collection was used. A portable benchtop centrifuge (Hettich EBA 20) was used for the preparation of PRP. Two rounds of centrifugation were carried out at 2529 RPM (500 G) and 5304 RPM (2200 G), respectively, for 10 minutes each [10] (Figure 1). The platelets were filtered out as PRP and were activated by adding 50 µl of 10% CaCl_2_; 0.5 ml of activated platelet-rich plasma was then injected into the osteoarthritic knee joints. A 20 gauge lumbar puncture needle was used for PRP collection.

Animal model of osteoarthritis

All animal models were done on 16 male Sprague Dawley rats, age 12-16 weeks (National Institute of Health, Islamabad, Pakistan), with the consent of the animal ethical review committee of Army Medical College, Rawalpindi, Pakistan. The rats were housed in groups in well-ventilated cages, under a 12-hour light-dark cycle at a temperature range of 20°C to 26°C, having access to water and food ad libitum.

The rats were allowed to accustom for a week before the start of the experiment. Unilateral OA was induced by a single intra-articular injection of 2 mg of monosodium iodoacetate (StruChem San Diego, Labseeker Inc., San Diego, California) in the right knee joint of animals. Monoiodoacetate (MIA)-induced OA is an established model for arthritic changes in rats [11-12]. All intra-articular injections were given under chloroform anesthesia, with insulin syringes (Sy’ah Impex, Karachi, Pakistan) using a 24 g needle (Jiangsu Jichun Medical Devices Co., Ltd. China). No substance was introduced in the contralateral knee. Rats were then randomly allotted to either the treatment group A (n=8 rats) or the non-treatment group B (n=8 rats). The treated group (A) received a single 0.5 ml injection of activated PRP 18 days after the MIA injection. During the experimental period, the animals in the treated group (A) were able to move freely for feeding food pellets and drink water from the bottles at the top of their cages. Rats were euthanized at eight weeks after the administration of PRP and knee joints were collected for histological analysis.

The knee joint was decalcified using 5% nitric acid for three to five days and the excess of nitric acid was removed by rinsing under tap water. The samples were then fixed in 10% formalin and, later, samples were air-dried and dehydrated by dipping in increasing concentrations of ethanol, i.e. 70%, 80%, and 90%. Sagittal sections of the specimen were cut and embedded in paraffin. Five micrometers thick sections were cut using a rotary microtome (Leica RM 255, Leica Biosystems, Wetzlar, Germany).

**Figure 2:**
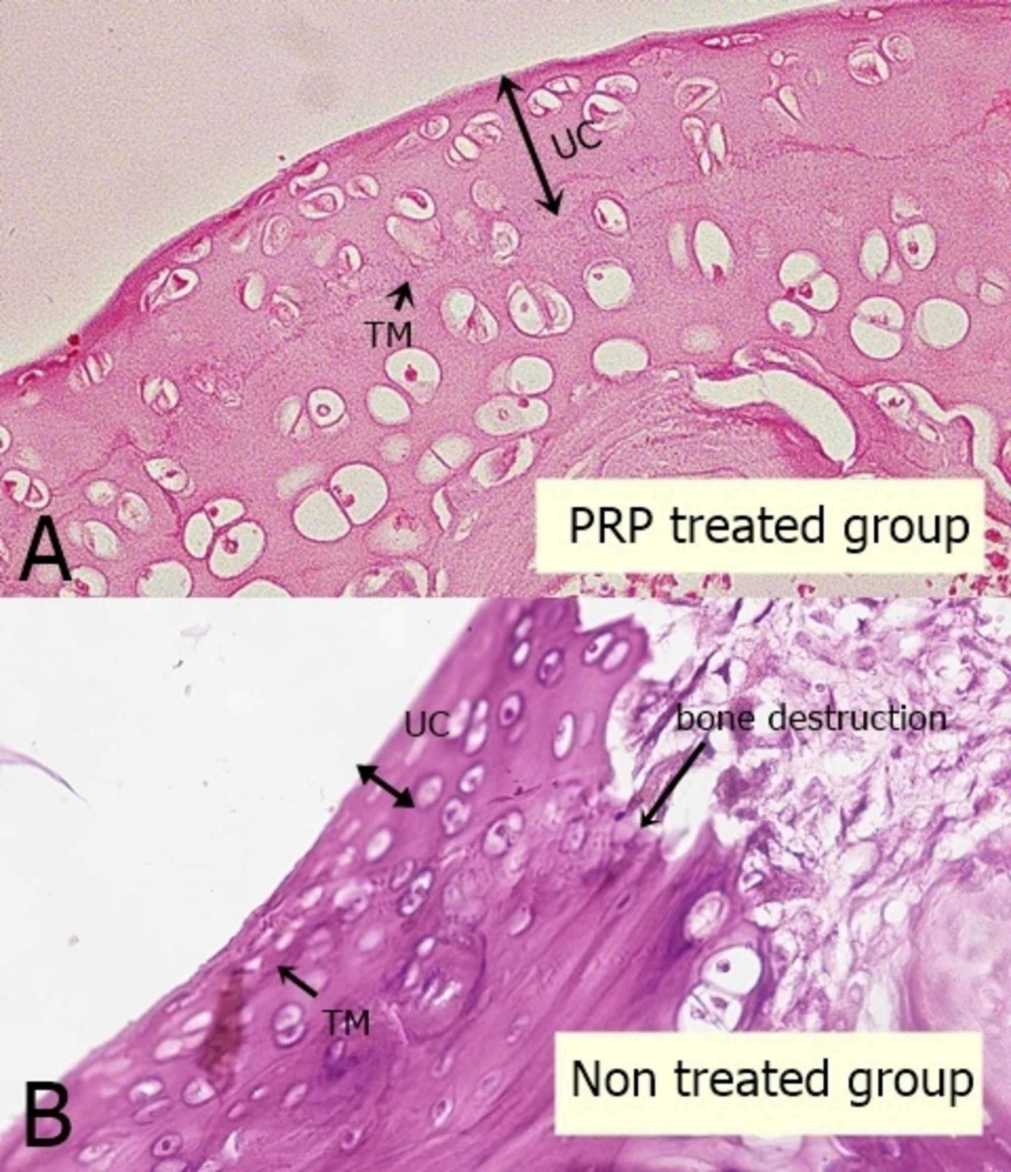
Microscopic picture of the articular cartilage of rat tibia showing the uncalcified cartilage thickness and changes in the chondrocyte morphology A: showing the cartilage of treated group having preserved cartilage height and active chondrocytes. B: showing the non-treated group with empty lacunae, severe decrease in cartilage thickness and bone destruction UC: uncalcified cartilage; TM: tidemark

The chondrocyte number and the thickness of the uncalcified cartilage were measured on H&E stained slides, 40X objective lenses were used, and the conversion factor was calculated for the eyepiece micrometer scale using a stage micrometer. Chondrocytes were counted in 0.06 mm^2 ^area calculated by micrometry. Four randomly selected sites, i.e. two from the periphery and two from the central zone of the cartilage, were chosen for calculating the thickness and number of chondrocytes, moving from the surface to the deeper zones up till the tidemark was reached (Figure 2) [13].

Data were entered and analyzed using SPSS version 21 (IBM Corp., Armonk, NY, US). Results were stated as means and standard deviations. Thickness was measured in micrometers for the individual specimen and the final average mean was calculated for the number of chondrocytes and the thickness of uncalcified cartilage. Images were taken from each section with the help of a camera (Olympus digital camera - 12 megapixel, Olympus Corporation, Tokyo, Japan) mounted on a microscope (Olympus DP22, Olympus Corporation. The mean and standard deviation was calculated for quantitative variables. The independent t-test was used for intergroup comparisons and the p-value was considered to be statistically significant at a cut-off value of ≤0.05.

## Results

Chondrocyte number

The mean chondrocyte number ± SD in the treated (A) and non-treated groups (B) was 38.12 ± 8.611 and 18.90 ± 13.8, respectively,(Table 1). The p-value for intergroup comparison was 0.006*, which was statistically significant.

**Table 1:** Table depicting mean and p-values for the thickness of uncalcified cartilage and the mean chondrocyte count among arthritic groups receiving PRP therapy (A) versus the non-treated group (B). PRP: platelet-rich plasma

Parameter	Treated Group A (Mean ± S.D)	Non-treated Group B (Mean ± S.D)	Intergroup comparison A vs B (p-value)
No. of normal chondrocytes	38.12 ± 8.611	18.90 ± 13.80	0.006*
Thickness of uncalcified cartilage (µm)	56.17 ± 16.34	23.78 ± 12.86	0.001*

Uncalcified cartilage thickness

The mean ± SD of uncalcified cartilage thickness of the treated group A and the non-treated group B was 56.17 ± 16.34 and 23.78 ± 12.86, respectively, which was statistically significant (p-value=0.001*) (Table 1).

## Discussion

Arthritis is a chronic inflammatory pathological condition having the characteristic features of pain, progressive cartilage degradation, and disability. Among the various therapeutic modalities being used, PRP has proven to reduce pain in patients with knee OA. IL-1β is believed to cause the degradation of the extracellular matrix along with the apoptosis of chondrocyte and tendon cells. IL-1β stimulates the release of many proteolytic enzymes and suppresses the synthesis of extracellular matrix proteins like aggrecan and collagen. In contrast to this, studies conducted on the chondrocytes of arthritic cartilage have shown that PRP plays an inhibitory role in matrix loss, and it is also evident in our study.

PRP, when injected into the body, is activated by thrombin or collagen, leading to the release of platelet-derived growth factors (PDGFs), tissue growth factors (TGFs), interleukin-1 receptor antagonist (IL-1RA), vascular endothelial growth factors (VEGFs), and fibrinogen. Studies regarding the role of growth factors present in PRP concluded that transforming growth factor-β (TGF-β) is one of the important growth factors responsible for the differentiation of mesenchymal stem cells (MSCs) and matrix deposition. TGF-β also enhances the expression of tissue inhibitors of metalloproteinases (TIMP-1). It is also believed to nullify the effects of inflammatory mediators, such as interleukins, and controls the metabolic functions of chondrocytes. The increase in chondrocyte proliferation and matrix deposition is said to be due to PDGFs, which are released by the α-granules of platelets. Moussa and his fellows studied the effects of PRP on the arthritic chondrocytes and concluded that chondrocyte propagation increases in a dose-dependent style in response to growth factors present in PRP. Moreover, chondrocytes senescence is reversed due to PRP-induced increase in autophagy [14]. Sengul and fellows studied the effect of PRP on rabbits by constructing the model of chest deformity and concluded that the administration of PRP is helpful in increasing the chondrocyte density and, consequently, results in an increased extracellular matrix [15]. These findings further strengthened our study, which showed a linear relationship between chondrocyte count and cartilage thickness.

The treated group receiving PRP therapy in our study had a higher chondrocyte number and more cartilage height as compared to the group that did not receive PRP. Yang and his colleagues carried out a study to see the effects of PRP on interleukin-1β-induced chondrocyte apoptosis and concluded that PRP administration significantly decreases the chondrocyte apoptosis and modified the apoptosis-associated expression at the level of genes [16]. This can be attributed to the presence of IL-1RA present in PRP, which inhibits the activation of the NFκB gene involved in apoptosis and inflammatory process [13]. In our study, we studied the effect of PRP on chondrocyte count in the arthritic rat knee cartilage and noted a decrease in the chondrocyte apoptosis in the PRP-treated group as evident by the higher chondrocyte number in the treated group A in comparison to the non-treated group B. As chondrocytes are the main cells secreting the matrix components, the protective role of PRP in chondrocyte apoptosis can be co-related to a lesser decrease in cartilage thickness noted in the PRP treated group of our study. Our results were further strengthened by studies, which showed a decrease in the rate of cartilage loss in some animal models treated with PRP [17]. Ornetti and his co-researchers concluded that Magnetic resonance imaging (MRI) in 73% of the arthritis patients receiving PRP treatment showed the absence of disease progression, which coincided with our study [9]. PRP response to articular cartilage thickness was also observed by other researches, which concluded that PRP did not enhance the cartilage thickness in 94% of the cases but 6% showed a minor enhancement. A review carried out by Fotouhi and his fellows concluded that PRP in combination with stromal vascular fraction (SVF) showed an extreme reduction in pain along with a gradual increase in the cartilage thickness at three and six months after the administration of injection [4].

The limitations of this study included the use of a single injection of PRP. Multiple injections would have provided strong evidence for the chondrogenic effect of PRP. This study is further limited by a lack of immunohistochemistry, which is required for the quantification of matrix loss.

## Conclusions

In this study, PRP administration halted the catabolic activity of chondrocytes, leading to a decreased rate of chondrocyte apoptosis, further resulting in less loss of the cartilage matrix secreted by cartilage cells. This conserved the cartilage height in the group receiving PRP, which showed that chondrocyte apoptosis directly affected the cartilage thickness in rat models of osteoarthritis. Therefore, it was concluded that PRP administration conserves the histomorphology of articular cartilage affected by osteoarthritis. To further establish the beneficial role of PRP, this study should be carried out using multiple injections of PRP.
